# Ferroelastic writing of crystal directions in oxide thin films

**DOI:** 10.1038/s41565-025-01950-z

**Published:** 2025-06-05

**Authors:** Wei Peng, Wenjie Meng, Younji Kim, Jiyong Yoon, Liang Si, Kesen Zhao, Shuai Dong, Yubin Hou, Chuanying Xi, Li Pi, Aditya Singh, Ana M. Sanchez, Richard Beanland, Tae Won Noh, Qingyou Lu, Daesu Lee, Marin Alexe

**Affiliations:** 1 Department of Physics, University of Warwick, Coventry, UK; 2 Center for Correlated Electron Systems, Institute for Basic Science, Seoul, Korea; 3 Department of Physics and Astronomy, Seoul National University, Seoul, Korea; 4 Anhui Key Laboratory of Low-Energy Quantum Materials and Devices, High Magnetic Field Laboratory, HFIPS, Chinese Academy of Sciences, Hefei, China; 5 Hefei National Research Center for Physics Sciences at the Microscale, University of Science and Technology of China, Hefei, China; 6 Department of Physics, Pohang University of Science and Technology, Pohang, Korea; 7 Institut für Festkörperphysik, TU Wien, Vienna, Austria; 8 School of Physics, Northwest University, Xi’an, China; 9 Anhui Laboratory of Advanced Photon Science and Technology, University of Science and Technology of China, Hefei, China

**Keywords:** Ferroelectrics and multiferroics, Magnetic properties and materials

## Abstract

Crystals often have complex structural domains, but a general method to remove or deterministically control such local heterogeneity is lacking. The resulting heterogeneity in crystal orientations obscures our understanding of material properties and can reduce the reliability and performance of related applications. Here, using shear stress from an atomic force microscope tip, we ferroelastically write local crystal orientations in oxide thin films. Applying this deterministic and reversible control to SrRuO_3_ and (La_0.7_Sr_0.3_)(Mn_0.9_Ru_0.1_)O_3_ films, we realize twin-free single crystals and design specific crystal-orientation domain textures at the nanoscale. Furthermore, through magnetoelastic coupling, we can mechanically manipulate the local magnetic anisotropy, and thereby write and erase functional nanoscale magnetic textures unattainable by conventional methods. Thus, pure mechanical force emerges as a means to control structural heterogeneity on demand and may make it possible to program electronic and spintronic functionalities.

## Abstract (web summary)

Local shear stress from an atomic force microscope tip can control the crystal directions in thin oxide films. This approach enables the manipulation of local magnetic anisotropy in ferromagnetic metals.

## Main

Ferroic crystals have switchable-order parameters, such as electric polarization, spin polarization and strain. Reversible and non-volatile control of local ferroic orders by their conjugate fields has been a key ingredient for various aspects of condensed matter physics and modern electronics. For instance, electric (magnetic) field switching of ferroelectric (ferromagnetic) dipoles has led to a variety of important technological applications such as memory devices and computing^1–5^. Recently, this control has reached a level where new types of dipole textures, including skyrmions and vortices, can be explored and manipulated^6,7^. These advances are expected to engender emergent quantum phenomena and functionalities for next-generation quantum devices.

Ferroelasticity—the largest class of ferroics—is characterized by an elastic hysteresis of switching between multiple orientation states (known as twins) of a crystalline lattice when this is subjected to mechanical stress^8^. In addition to its manifestations in a range of crucial mechanical properties such as the shape memory effect and superelasticity^9–11^, ferroelasticity can also modulate electronic properties through the coupling of structural and electronic degrees of freedom. Specifically, formation of ferroelastic domains leads to real-space structural heterogeneity, impacting materials anisotropy^12–15^ and introducing two-dimensional topological defects, that is, domain walls^16–19^. A recent study of moiré superlattices further revealed that electronic interactions and band structure can be engineered by crystalline rotation of a superlattice^20^—a process in which ferroelasticity can play a role. However, unlike its electric and magnetic counterparts, there has been a lack of viable means to deterministically and reversely control local ferroelastic orders. This greatly hinders our understanding and optimization of material properties and related applications, particularly in terms of orientation heterogeneity. It also impedes further implementation of ferroelastic order parameters as an effective tuning mechanism for functional electronic materials.

In this paper, we exploit local stress generated by an atomic force microscope (AFM) tip for controlling local ferroelastic orders. Previous work has shown the capability of an AFM tip for mechanical switching of ferroelectric polarization, although this relies on the coupling between polarization and a strain gradient^21^ and has only been demonstrated for insulators. Here we show that the tip-induced stress can be utilized to directly switch ferroelastic orders and associated crystal orientation states of metallic oxide thin films. Specifically, in two representative ferromagnetic metals, SrRuO_3_ and (La_0.7_Sr_0.3_)(Mn_0.9_Ru_0.1_)O_3_, we achieved ferroelastic writing of nanoscale crystal-orientation domain textures with depth controllability, observed by scanning electron microscopy (SEM). Numerical analysis combined with experimental examination of tip-scanning parameters suggests that shear stress plays a critical role in the switching process. Furthermore, by magnetotransport measurements and magnetic force microscopy (MFM), we demonstrate ferroelastic control of the local magnetic anisotropy through magnetoelastic coupling, realizing functional nanoscale magnetic textures on demand.

## Ferroelastic orders in oxides

**Fig. 1:**
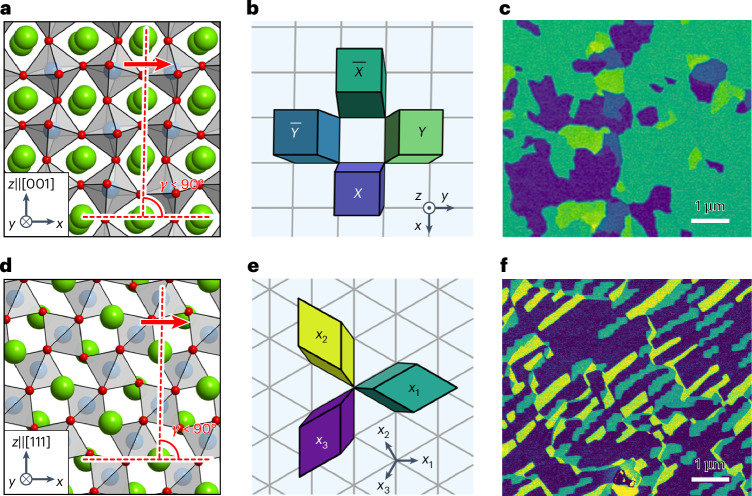
Ferroelastic domains in perovskite oxides. **a**, Atomic structure in side view of (001)-oriented perovskite oxides with orthorhombic or monoclinic structure. The lattice shears towards the *x* direction (red arrow), namely [100]. **b**, Top-view schematic of four different ferroelastic domains for (001)-oriented monoclinic or orthorhombic perovskites. *X*, $\overline{X}$, *Y* and $\overline{Y}$ indicate the ferroelastic domains shearing along the *x*, −*x*, *y* and −*y* directions, respectively. **c**, ECC image of as-grown SrRuO_3_ (001) thin films. The image was processed with false colours following the colour code in **b**. **d**, Atomic structure in side view of (111)-oriented perovskite oxides in the monoclinic structure. The lattice shears towards the *x* direction (red arrow), namely $[11\overline{2}]$. **e**, Top-view schematic of three-variant ferroelastic domains for (111)-oriented perovskite oxides. *X*_1_, *X*_2_ and *X*_3_ indicate the ferroelastic domains shearing along one of the equivalent $\langle 11\overline{2}\rangle$ directions (marked as *x*_1_, *x*_2_ and *x*_3_). **f**, ECC image of as-grown SrRuO_3_ (111) thin films, which was processed with false colours following the colour code in **e**.

SrRuO_3_ and (La_0.7_Sr_0.3_)(Mn_0.9_Ru_0.1_)O_3_ at room temperature adopt low-symmetry (orthorhombic and monoclinic) crystal structures, depending on the conditions of material fabrication^22,23^. As shown in Fig. 1a,d, the structural distortion in these oxides manifests as lattice shearing from the ideal cubic unit cells, which is a general characteristic for many other low-symmetry structures. When deposited as a heterostructure on higher-symmetry single-crystal substrates, the oxides can exhibit degenerate orientation states characterized by the shear direction. For instance, (001)_pc_-oriented (the subscript pc refers to pseudocubic and is omitted for simplicity hereinafter) SrRuO_3_ and (La_0.7_Sr_0.3_)(Mn_0.9_Ru_0.1_)O_3_ films on (001) cubic substrates are expected to possess four ferroelastic domain variants (Fig. 1b), while there are three such variants for (111)-oriented SrRuO_3_ (Fig. 1e)^22^.

To probe the ferroelastic domains, we applied the technique of electron channelling contrast (ECC) imaging by SEM. The contrast in this imaging mode is given by the electron channelling probability, which is sensitive to the lattice orientation^24^. We carried out the ECC imaging for as-grown SrRuO_3_ films on SrTiO_3_ (001) and (111) substrates (Fig. 1c,f, respectively), and (La_0.7_Sr_0.3_)(Mn_0.9_Ru_0.1_)O_3_ films on (LaAlO_3_)_0.3_(Sr_2_TaAlO_6_)_0.7_ (001) substrates (Supplementary Fig. 1). The images display distinct levels of channelling contrast, corresponding to the expected domain degeneracy.

## Local crystal direction control via ferroelastic writing

**Fig. 2:**
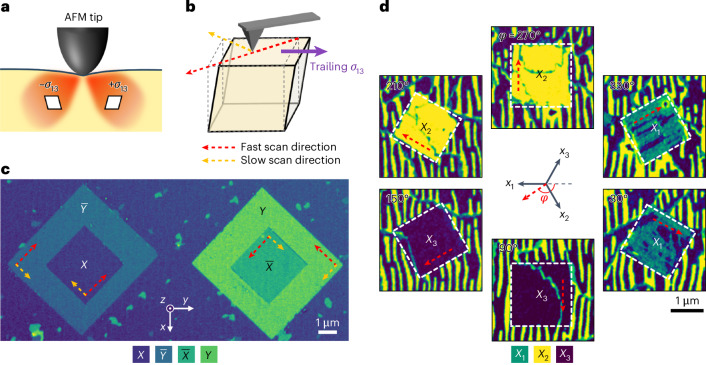
Ferroelastic writing and crystal direction selection. **a**, Schematic distribution of the shear stress *σ*_13_ under the AFM tip loading. There are two lobes for positive or negative shear stresses, as indicated by the rhombus symbol. **b**, Schematic of crystal shearing by the trailing *σ*_13_ according to the fast and slow scan axes. **c**,**d**, ECC images demonstrating deterministic ferroelastic writing, corresponding to four-variant (**c**) and three-variant (**d**) crystal direction selection in SrRuO_3_ (001) and (111) thin films, respectively. *X*, $\overline{X}$, *Y* and $\overline{Y}$ indicate the ferroelastic domains shearing along *x*, −*x*, *y* and −*y* directions, respectively, for SrRuO_3_ (001). *X*_1_, *X*_2_ and *X*_3_ indicate the ferroelastic domains shearing along one of the equivalent $\langle 11\overline{2}\rangle$ directions (marked as *x*_1_, *x*_2_ and *x*_3_) in SrRuO_3_ (111).

**Extended Data Fig. 1:**
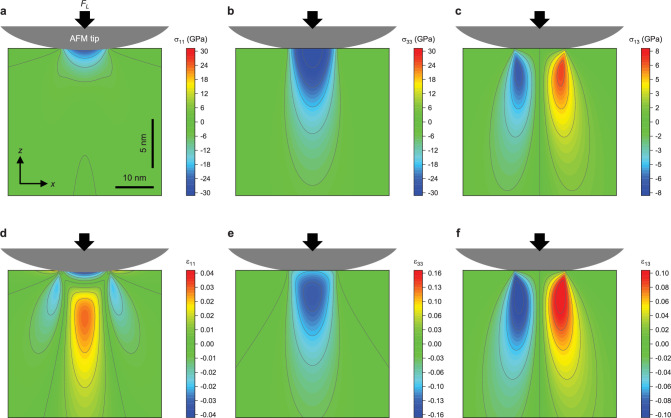
Stresses and strains calculated for a static AFM tip using the Hertzian contact model. Spatial distributions of the stresses *σ*_11_ (**a**), *σ*_33_ (**b**), and *σ*_13_ (**c**), and the strains *ε*_11_ (**d**), *ε*_33_ (**e**), and *ε*_13_ (**f**), calculated with the parameters *F*_*L*_ = 3 μN and *R* = 20 nm. Source data

**Extended Data Fig. 2:**
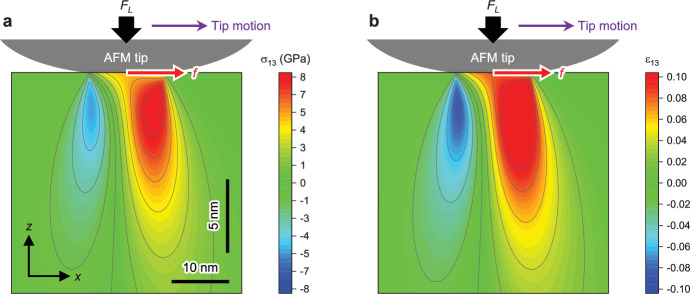
Stresses and strains calculated for a sliding AFM tip using the Hertzian contact model. Spatial distributions of the shear stress *σ*_13_ (**a**) and the shear strain *ε*_13_ (**b**) under a sliding AFM tip with a loading force *F*_*L*_ of 3 μN and a tangential frictional force *f*. The calculations were performed using a friction coefficient of *k*_*f*_ = 0.15. The value of *k*_*f*_ between a diamond tip and a well-polished metallic material is known to range from 0.1 to 0.15. Source data

Normally, the ferroelastic domain textures in metallic oxides do not respond to typical excitations, such as electric and magnetic fields, nor can they be deterministically controlled by a conventional axial stress field. Instead, we exploit the symmetry-breaking mechanical stress generated by an AFM tip to switch the ferroelastic domains. Under the application of a normal loading force, an AFM tip generates a complex (Hertzian) stress field in the vicinity of the tip–film contact area^25^. Of particular interest is an antisymmetric shear stress *σ*_13_ (Fig. 2a and Supplementary Fig. 2), which is capable of switching the lattice-sheared ferroelastic domains. Detailed analysis of the stress and strain distributions can be found in Methods and Extended Data Figs. 1 and 2. We find that, by sliding the tip in a direction along, or close to, the desired lattice shear direction, it is possible to switch the scanned region using the net trailing component of *σ*_13_ (Supplementary Fig. 3).

Based on this, we demonstrated local ferroelastic writing and deterministic crystal direction selection for SrRuO_3_ (001) (Fig. 2c), SrRuO_3_ (111) (Fig. 2d and Supplementary Fig. 4) and (La_0.7_Sr_0.3_)(Mn_0.9_Ru_0.1_)O_3_ (001) films (Supplementary Fig. 1). We carried out the ECC imaging after mechanical writing by an AFM tip. The ECC images clearly evidence the local switchability of ferroelastic domains by the tip. The tip scan direction deterministically controls the ferroelastic domains and associated crystal orientations of the scanned region, in line with the proposed mechanism based on the trailing *σ*_13_. Furthermore, the ferroelastic writing exhibits excellent reproducibility, across a wide range of the tip scan directions. Crystal direction selection of the four- and three-variant systems is allowed by the net trailing *σ*_13_ within angles of around ±45° and ±60° with respect to the lattice shear direction, respectively. Due to such a high tolerance of tip movement, tip-induced mechanical switching offers a practical technique for controlling the ferroelastic domains and associated crystalline directions.

**Extended Data Fig. 3:**
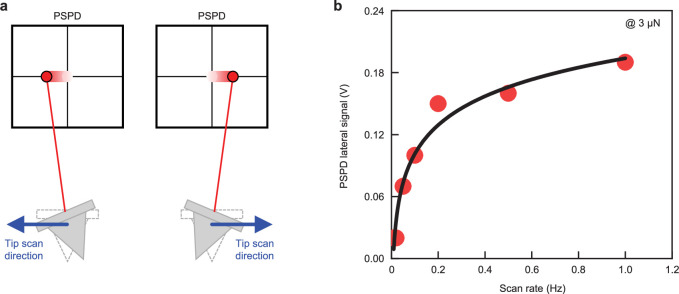
Characterization of friction for a sliding AFM tip. **a**, Schematic illustration of lateral photodiode signals of an AFM tip induced by friction during scanning. **b**, Position sensitive photodiode (PSPD) lateral signals measured as a function of AFM tip fast-scan rate. The black solid curve shows a logarithmic fit. The tip loading force was fixed at 3 μN. The considerably reduced PSPD signal at a low scan rate of 0.01 Hz indicates sufficiently suppressed friction. Source data

**Extended Data Fig. 4:**
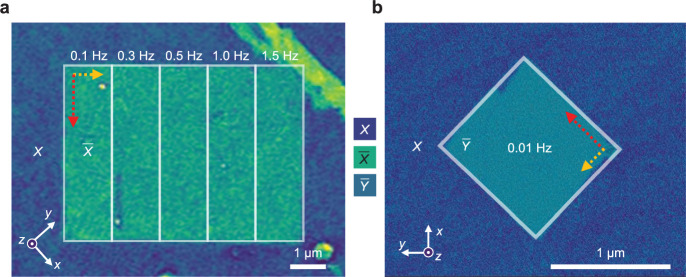
Dependence of ferroelastic writing on scan rate. ECC images of ferroelastic domains written at fast-scan rates of 0.1–1.5 Hz (**a**) and 0.01 Hz (**b**) in SrRuO_3_ (001) thin films. The white boxes mark the domains written at different fast-scan rates. Red and yellow dotted arrows indicate the fast and slow scan directions. The tip loading force was fixed at 3.2 μN.

It is important to note that friction is supposed to accompany tip scanning in addition to the Hertzian stress field, and may also contribute to the ferroelastic switching. To examine its contribution, we performed ferroelastic writing with varying scan rates. As the friction varies logarithmically with the scan rate^26^, it can be largely suppressed at an extremely low rate, for example, 0.01 Hz (Extended Data Fig. 3). The successful ferroelastic switching irrespective of scan rate shown in Extended Data Fig. 4 thus underscores the critical role of static shear stress rather than friction.

**Extended Data Fig. 5:**
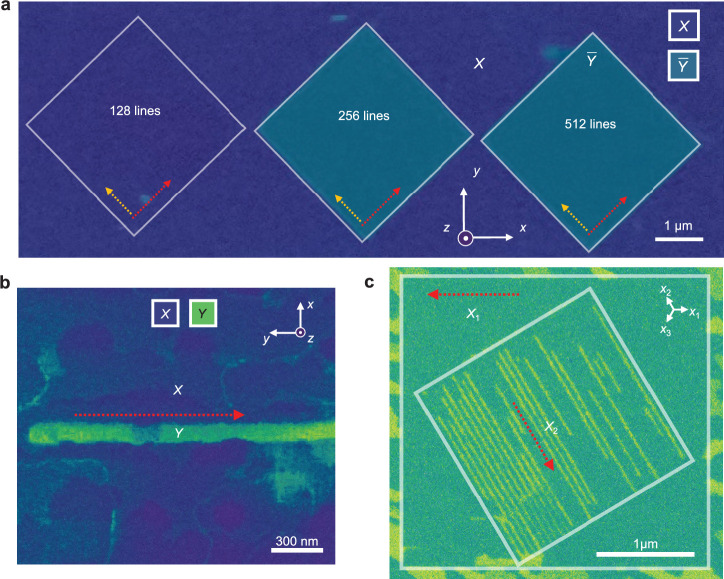
Dependence of ferroelastic writing on scan resolution. **a**, ECC image of ferroelastic domains written with different scanning resolutions of 128, 256, and 512 lines in a 10 nm-thick SrRuO_3_ (001) thin film. The white boxes mark the ferroelastically written regions. Red and yellow dotted arrows indicate the fast and slow scan directions. The tip loading was fixed at 3.4 μN. **b**, ECC image of SrRuO_3_ (001) showing a stripe domain with a width of approximately 100 nm, written by a tip with a radius of approximately 100 nm and a relatively high loading force of 7.5 μN. **c**, ECC image of SrRuO_3_ (111) showing stripe domains with a width of approximately 20 nm. The outer box of 3 × 3 μm^2^ area was written in the *X*_1_ direction with 256 lines, and the line domains were written with 30 lines in the *X*_2_ direction for a 2 × 2 μm^2^ area. A loading force of 3.0 μN was used for both areas. Fast scan directions are marked by red arrows.

The effects of scan resolution are also examined. Although writing with dense scanning lines (namely high resolution) always leads to ferroelastically switched regions, sparse writing fails in SrRuO_3_ (001) films (Extended Data Fig. 5a). We suppose that this is related to domain stability, which is determined by the domain-wall energy and domain size. As a switched stripe region is obtained by scanning along each line path, a low scan resolution leads to isolated stripe domains. These domains may not be sufficiently persistent for ECC imaging (with a lapse of time around several hours) due to their narrow width (<20 nm according to the contact radius). Indeed, a single stripe domain with its width increased to approximately 100 nm is stabilized (Extended Data Fig. 5b). In contrast, stripe domains with a width of approximately 20 nm can be stabilized in SrRuO_3_ (111) films (Extended Data Fig. 5c). The difference may be a result of different domain-wall energies and the different numbers of ferroelastic variants in the two systems, which requires further investigation.

## Control of local ferromagnetic anisotropy

The heterogeneity of crystal orientations in ferromagnetic oxides leads to substantial magnetic inhomogeneity due to their magnetoelastic anisotropy^27,28^, which was found to sustain an incredibly large magnetic field up to 27 T in SrRuO_3_ (111) films (Supplementary Figs. 5–7). Such a robust anisotropy renders the control and elimination of magnetic inhomogeneity through the use of magnetic field challenging and impractical. Instead, we show that ferroelastic switching is an effective approach to manipulate such local magnetic anisotropy.

**Extended Data Fig. 6:**
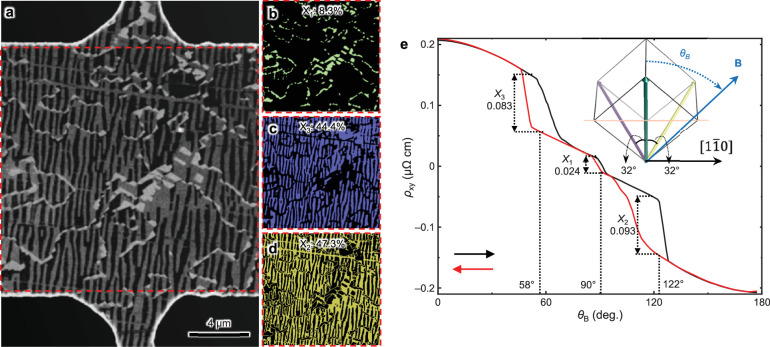
Statistics of ferroelastic domain populations and analysis of magnetic anisotropy for SrRuO_3_ (111). **a**, Image thresholding analysis of the ECC image of the Hall bar device of SrRuO_3_ (111) to estimate its ferroelastic domain populations (**b**–**d**), which are 8.3% (*X*_1_), 44.4% (*X*_3_), and 47.3% (*X*_2_), respectively. **e**, Transverse resistivity of the device in (**a**) with the magnetic field rotating in the $(11\overline{2})$ plane, displaying three hysteretic jumps due to magnetic switching. The inset schematic illustrates the geometric relation between the inferred MEAs and the crystalline directions in the projected $(11\overline{2})$ plane. Thus, the MEAs are supposed to along one of the three <110> directions tilted from the out-of-plane direction. This is also corroborated by the measurement in the $(1\overline{1}0)$ plane in Extended Data Fig. 7. Accordingly, the magnetic populations are estimated to be 46.5% (*X*_2_), 41.5% (*X*_3_), and 12% (*X*_1_), based on the change of the Hall resistivity. The black and red arrows indicate the field scanning directions. Source data

**Extended Data Fig. 7:**
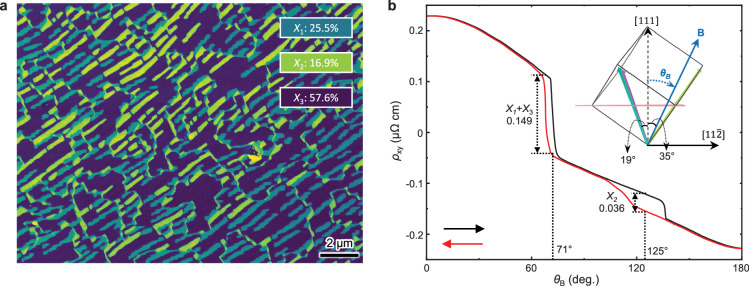
Statistics of ferroelastic domain populations and analysis of magnetic anisotropy for SrRuO_3_ (111) using a different sample. **a**, ECC image to show the ferroelastic domain structure of SrRuO_3_. By image thresholding analysis, the domain populations are estimated to be 57.6% (***X***_**3**_), 25.5% (***X***_**1**_), and 16.9% (***X***_**3**_), respectively. **b**, Transverse resistivity versus the magnetic field angle, showing two hysteretic jumps. As shown by the inset schematic, the magnetic easy axes <110> of two ferroelastic domains form the same angle, ~19°, with the out-of-plane [111] axis in the projected $(1\overline{1}0)$ plane, while it is ~35° for the third domain. This agrees with the observed two hysteretic jumps in the angle-dependent transverse resistivity. Accordingly, the magnetic populations are estimated to be 80.5% and 19.5%, which correspond well to the ferroelastic domains (***X***_**1**_ + ***X***_**3**_ = 83.1%) and (***X***_**2**_ = 16.9%), respectively. Source data

We first performed angle-dependent anomalous Hall effect (AHE) measurements to examine the magnetic anisotropy and magnetoelastic coupling. In a ferromagnetic material with large magnetic anisotropy, a rotating magnetic field *B* is able to induce abrupt 180° magnetization reversal along the magnetic easy axis (MEA) when the *B* field rotates by over 90° from its initial magnetization direction. The reversal process induces a hysteretic jump of the anomalous Hall resistivity *ρ*_AHE_, determined by the equation *ρ*_AHE_ = *R*_S_*M* = *R*_S_d*m*/d*V*, where *R*_S_, *M*, *m* and *V* indicate the AHE coefficient, magnetization, total magnetic moments and volume, respectively. Therefore, we can determine the orientation of MEAs, and the variation of *ρ*_AHE_ can be used to estimate the volume of corresponding magnetic domains that undergoes magnetization reversal. As shown in Extended Data Figs. 6 and 7, SrRuO_3_ (111) exhibits three MEAs aligning along the pseudocubic face diagonal, namely <110>, and the corresponding domain populations can be extracted. We also estimated the populations of ferroelastic domains by thresholding analysis of the ECC images, which, as expected, match those of the magnetic species. This result implies that the magnetic anisotropy is tied to the crystal orientation via magnetoelastic coupling.

**Fig. 3:**
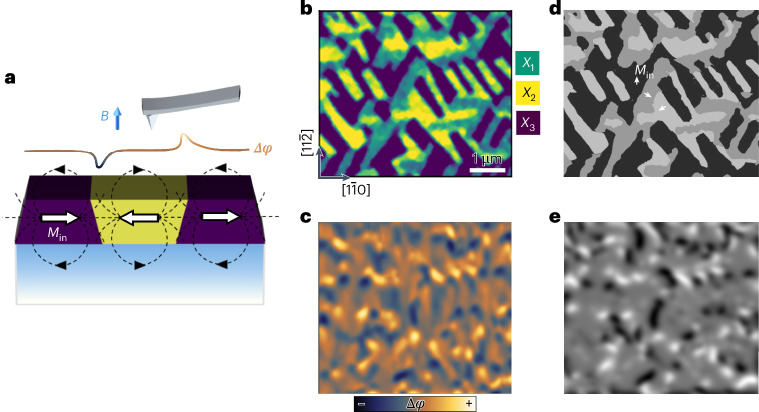
Ferroelastically coupled ferromagnetic domains. **a**, Schematic to show the emergence of magnetic contrast in MFM (reflected from the phase shift Δ*φ* of the tip resonance) induced by the stray fields at the domain walls. The stray fields are related to specific configurations of in-plane magnetization components (*M*_in_), forming either head-to-head or tail-to-tail patterns. **b**,**c**, ECC image (**b**) and MFM image (**c**) of SrRuO_3_ (111) film captured from the same region at 300 and 5 K, respectively. **d**, Binary image of the corresponding magnetic domain textures constructed from the ferroelastic domain structure in **b**, based on the magnetic anisotropy of each ferroelastic variant along <110>. The white arrows indicate the *M*_in_ of each domain species. **e**, Simulated MFM image of the magnetic domains in **d** using a micromagnetic simulator, which matches well the experimental image in **c**. The images share the same scale bar as in **b**.

We used MFM to further confirm the magnetoelastic coupling. Due to the tilted geometry of MEAs in SrRuO_3_ (111), the in-plane magnetization components (*M*_in_) will arrange in head-to-head or tail-to-tail patterns at the domain walls, leading to opposite stray fields (Fig. 3a). Therefore, even the out-of-plane magnetization is essentially uniform across ferroelastic domains under poling, and these domain-wall stray fields can cause sizeable magnetic contrast in the MFM images, offering a way to examine the magnetic domains. Figure 3b,c shows the ECC and MFM images of the same region. Based on the inferred MEAs, the magnetic domain textures corresponding to the ferroelastic domains can be constructed as in Fig. 3d. Accordingly, an MFM image can be simulated according to the distribution of stray fields (Fig. 3e), which matches the experimental image.

**Fig. 4:**
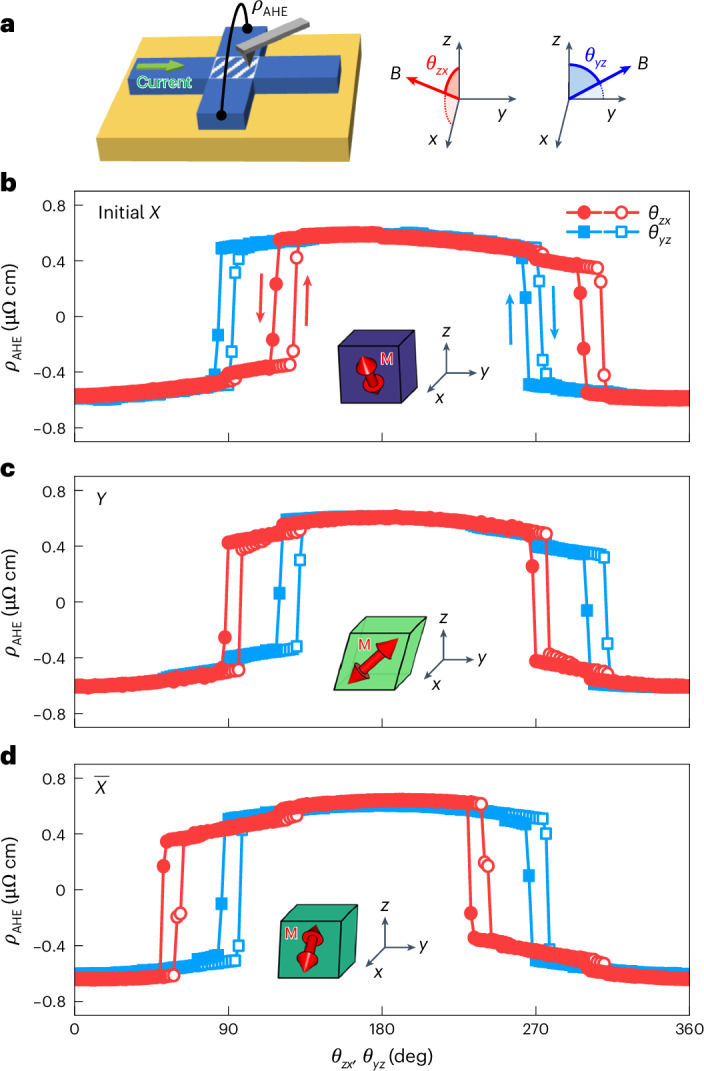
Crystal-direction-selection-based control of ferromagnetic anisotropy. **a**, Schematic of the Hall bar device of SrRuO_3_ (001). After ferroelastic writing by an AFM tip, the anomalous Hall resistivity *ρ*_AHE_ was measured for a range of directions. *θ*_*zx*_ and *θ*_*yz*_ indicate the rotation angle of the applied *B* field on the *zx* and *yz* planes, respectively. **b**–**d**, *ρ*_AHE_ measured at the initial state with dominant *X* domains (**b**), after being mechanically switched to the *Y* domains (**c**) and consecutively switched to $\overline{X}$ domains (**d**) with the same Hall bar device. The measurements were performed at *T* = 70 K and *B* = 1.6 T while scanning both *θ*_*zx*_ and *θ*_*yz*_ to unambiguously determine the magnetic anisotropy. Insets: schematic illustrations of the orientation of ferroelastic domains and ferromagnetic anisotropies (red arrows). Source data

**Extended Data Fig. 8:**
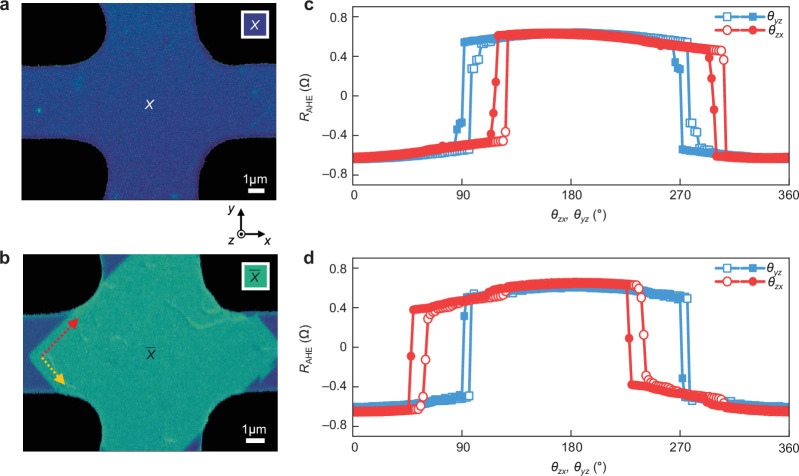
Control of crystal directions and ferromagnetic anisotropy on a Hall bar device of SrRuO_3_ (001). **a**, **b**, ECC image of a 10 nm-thick SrRuO_3_ (001) Hall bar device with *X* (**a**) and $\overline{X}$ (**b**) domains. The device was written with a loading force of 3.0 μN, with red and yellow arrows depicting fast and slow scan directions. **c**, **d**, Angle-dependent AHE measurement results taken from the Hall bar device shown in (**a**) and (**b**), respectively. The definition of *θ*_*zx*_ and *θ*_*yz*_ are the same as in Fig. 4.

The above results suggest that ferroelastic switching should be naturally accompanied with altered MEAs. To confirm this, we controlled the crystal orientations of SrRuO_3_ (001) films via ferroelastic writing and then identified the changed MEAs by measuring *ρ*_AHE_ (Fig. 4a). Figure 4b, Extended Data Fig. 8 and Supplementary Fig. 8 highlight that the MEAs of SrRuO_3_ can be controlled deterministically and reversibly via ferroelastic writing. The MEAs in SrRuO_3_ (001) were found to tilt by around 30° from the *z* axis toward the lattice shear direction (for example, the *x* direction for the *X* domain). Consistent with this correlation, our angle-dependent AHE measurements revealed a precisely controlled MEA by ferroelastic writing (Fig. 4b–d). Similar results for (La_0.7_Sr_0.3_)(Mn_0.9_Ru_0.1_)O_3_ (001) films can be found in Supplementary Fig. 9.

**Extended Data Fig. 9:**
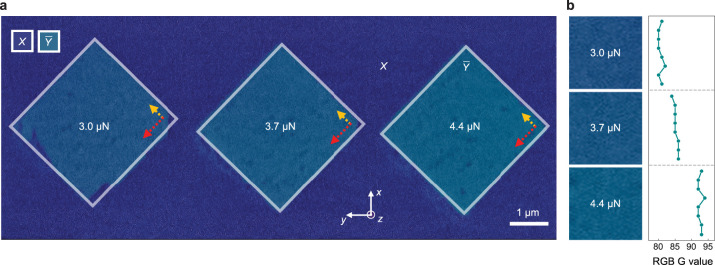
Partial ferroelastic writing along the depth direction examined by ECC imaging. **a**, ECC image of ferroelastic domains written at different loading forces of 3.0, 3.7, and 4.4 μN in a 20 nm-thick SrRuO_3_ (001) thin film. The white boxes mark the ferroelastically written regions. Red and yellow dotted arrows indicate the fast and slow scan directions. **b**, To clearly visualize the colour differences in the ECC image, the three regions written with different loading forces in (**a**) are displayed as 1 × 1 μm^2^ cropped images. As the loading force increases, gradual increment of the G value in the ECC colour (right panel) suggests increasing switched volume.

**Extended Data Fig. 10:**
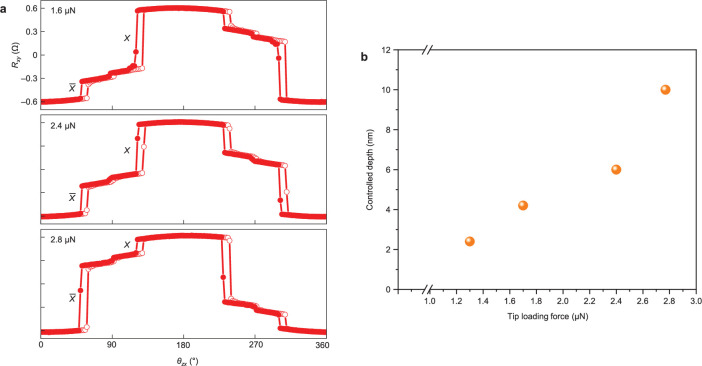
Partial ferroelastic writing along the depth direction examined by AHE measurements. **a**, Angle-dependent AHE measurements for a 10 nm-thick SrRuO_3_ (001) film after ferroelastic writing with varying loading forces. The SrRuO_3_ (001) film initially possessed dominant *X* domains. With intermediate loading force, the X domains were partially switched to $\overline{X}$ domains. The proportion of switched volume increases with the loading force, corresponding to the thickness being switched. The SrRuO_3_ was capped with 4 unit cell-thick SrTiO_3_. **b**, Controlled depth as a function of AFM tip loading forces. Source data

Additionally, varying the tip loading force allows for systematic control of the depth of ferroelastic switching, based on the spatial confinement of tip-induced mechanical stresses. While a loading force of approximately 3 μN can control the crystal orientation across almost the entire film thickness of around 10 nm, a reduced loading force selectively targets the orientation of the upper region. This enables depth control of crystal orientations with nanometre-scale resolution, as confirmed by a fractional change of *ρ*_AHE_ and by intermediate ECC with a reduced loading force (Extended Data Figs. 9 and 10 and Supplementary Fig. 10). Our ferroelastic writing therefore offers a method for selecting crystal directions and associated MEAs in a three-dimensional manner with complete controllability both laterally and over the depth of the film.

**Fig. 5:**
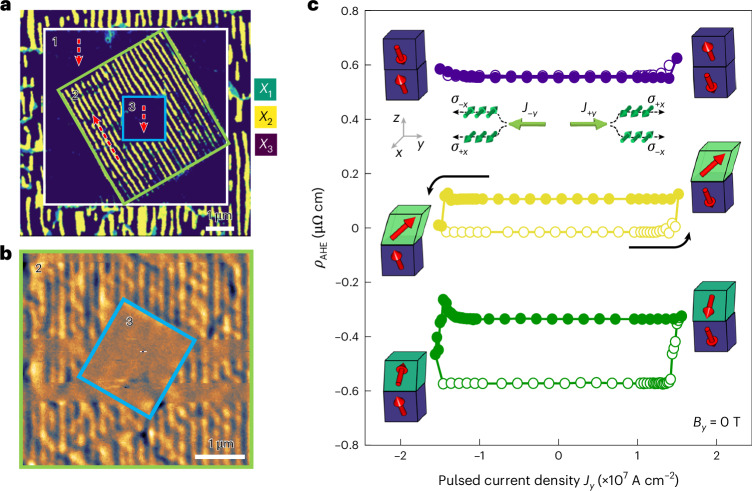
Crystal-direction-selection-based control of local magnetic textures and functionalities. **a**, ECC image of mechanically written ferroelastic domain textures in SrRuO_3_ (111). After writing a homogeneous *X*_3_ domain in the white-box region (1), *X*_2_ stripe domains were written in the green-box region (2). Then, a homogeneous *X*_3_ domain is overwritten in the blue-box region (3). Red dashed arrows indicate the AFM-tip scan directions. **b**, MFM image showing the ferromagnetic domain textures of regions 2 and 3 in **a** obtained at 100 K. **c**, Field-free SOT switching loops of SrRuO_3_ (001) with differently designed vertical heterogeneities of ferromagnetic anisotropy (shifted for better visibility). The SOT switching experiments were performed using the same Hall bar device at 70 K without an external magnetic field (that is, *B*_*y*_ = 0 T). The black arrows indicate the current sweeping directions. The red arrows in the inset schematic illustrate the magnetization direction after current-induced SOT switching. The change in *ρ*_AHE_ corresponds to the extent of perpendicular magnetization induced by SOT switching. Source data

Deterministic control of local crystal orientation and magnetic anisotropies via ferroelastic switching is able to unlock possibilities for engineering magnetic and electronic properties in situations where an electric or magnetic field works inefficiently. Using the SrRuO_3_ (111) film as an example, by performing a sequence of mechanical switching along different scan paths, we could selectively erase and rewrite ferroelastic nanodomains in the form of stripes approximately 50 nm wide (Fig. 5a). Using a sharper tip, we managed to further acquire dot domains of 26 × 26 nm^2^ in size (Supplementary Fig. 11). Consequently, the ferroelastically written nanodomains lead to readable magnetic states, as shown in Fig. 5b and Supplementary Fig. 12. These elastically stabilized magnetic states point to a prototypical magnetic memory that can be mechanically programmed with nanoscale precision and exhibits extraordinary robustness (Supplementary Figs. 5–7), in contrast to conventional magnetic storage media.

Beyond the ability to write lateral magnetic textures, we further exploited the depth controllability of the tip-induced ferroelastic switching. This depth control unlocks the possibility of designing vertical heterogeneity in magnetic anisotropy, which could be used to develop electronic and spintronic applications. In particular, we show that this could facilitate mechanically tunable field-free spin–orbit torque (SOT) switching of perpendicular magnetization in a single-layer ferromagnet, such as SrRuO_3_ (Fig. 5c and Supplementary Fig. 13; see also Methods). SrRuO_3_ simultaneously exhibits metallicity, ferromagnetism and a substantial spin Hall effect, enabling its application as a single-layer SOT switching device^29,30^. The spin Hall effect typically generates spin accumulations of equal magnitude but opposite directions at both surfaces (Fig. 5c, inset), preventing net SOT magnetization switching within a single layer. As a result, SrRuO_3_ (001) films in a single-domain state did not show net switching (purple circles in Fig. 5c). However, when we ferroelastically switched the top region and its MEA, the spin Hall effects at the bottom and top layers no longer cancelled each other out, yielding net SOT magnetization switching without an external magnetic field^28^. Interestingly, depending on the crystal rotation angle, we could also control the extent of partial magnetization switching. When the top layer was rotated by 180°, the change of *ρ*_AHE_ due to SOT magnetization switching doubles (green circles in Fig. 5c) compared with when the top layer was rotated by 90° (yellow circles in Fig. 5c). This is because SOT switching in the 180°-rotated top layer is an addition to that in the bottom layer, whereas the 90°-rotated top layer does not contribute to deterministic SOT switching.

## Conclusions

Our work demonstrates three-dimensional ferroelastic writing for deterministic and reversible control of local crystal orientations via an AFM tip. Although ferroelectric and ferromagnetic order parameters are often mutually exclusive, ferroelasticity is compatible with every other important property such as polarity, metallicity and magnetism. This technique will therefore advance the study and control of a rich spectrum of structural-heterogeneity-relevant properties, such as superconductivity^12,31^, magnetotransport^32,33^, multiferroics^7,34^, photoconductivity^14^ and domain-wall-coupled emergent phenomena^17,19^. For example, we have already shown its application in mechanically programmable magnetic memories and spintronics by manipulating the local magnetic anisotropy in a ferromagnetic metal. The density of such a prototypical memory reaches 148 Gbit cm^–2^ (estimated from a domain size of 26 × 26 nm^2^ in Supplementary Fig. 11) with unprecedented stability. Moreover, inspired by the recent invention of twistronics, interdomain electronic interactions at the heterointerface of different orientation states are attracting ever-increasing interest^35,36^. In particular, the relevant physics can be also explored in the realm of oxides by taking advantage of recent advancements of large-scale free-standing membranes^37^. These studies will be greatly accelerated by the current technique of controlling local structural orientations. A class of mechanically programmable, non-volatile nanoelectronics, in which the orientation states play a key role, is thereby envisioned.

## Methods

### Sample growth

SrRuO_3_ films were grown on SrTiO_3_ (001) and (111) substrates at 680 °C in a pulsed-laser deposition system equipped with a KrF excimer laser (248 nm). The growth was performed under an oxygen pressure of 100–120 mtorr with a laser fluence of 1.5–2.0 J cm^−2^. A high-pressure reflection high-energy electron diffraction system was used to monitor the growth. The thickness of films in the work was around 10 nm. Before deposition, SrTiO_3_ substrates were etched with a buffered hydrofluoric acid solution and then annealed in air at 1,000 °C for 3 h to produce an atomically flat surface with a unit-cell step terrace structure. (La_0.7_Sr_0.3_)(Mn_0.9_Ru_0.1_)O_3_ films were grown on (LaAlO_3_)_0.3_(Sr_2_TaAlO_6_)_0.7_ (001) substrates at 750 °C under an oxygen pressure of 225 mtorr with a laser fluence of 2.0 J cm^−2^. Before deposition, (LaAlO_3_)_0.3_(Sr_2_TaAlO_6_)_0.7_ substrates were cleaned with deionized water and acetone then annealed in air at 1,050°C for 6 h to obtain an atomically flat surface.

### SEM

A Zeiss Gemini or JEOL SEM with a back-scattered electron detector was used to capture the ECC images of the SrRuO_3_ and (La_0.7_Sr_0.3_)(Mn_0.9_Ru_0.1_)O_3_ films. To acquire sufficient signal from the ultrathin oxide films, a high gain was applied, and a 60-μm aperture in the high-current mode was used for the incident electron beam at 9–10 kV. The sample was tilted by 1°–5° and rotated in the plane to achieve the best contrast for all the domain species. The ECC images were processed in false colours with the Gwyddion image-processing package.

### Device fabrication and transport measurement

Conventional photolithograph and solvent etching by NaIO_4_ solution (0.1 M) were used to pattern the SrRuO_3_ (111) films into the Hall bar geometry. For SrRuO_3_ (001) and (La_0.7_Sr_0.3_)(Mn_0.9_Ru_0.1_)O_3_ (001) films, conventional photolithography and ion beam milling were used to pattern Hall bar geometry. The transverse resistivity was measured with a physical properties measurement system (Quantum Design).

### MFM and micromagnetic simulations

MFM measurements were carried out using a custom-built, variable-temperature, high-magnetic field set-up, incorporating a 12-T superconducting magnet. For higher-field measurements, a specialized MFM system was used, featuring a 35-T water-cooled magnet from the Chinese High Magnetic Field Laboratory, Hefei. The force sensor was a commercial piezoresistive cantilever (PRSA-L300-F50-STD from SCL-Sensor Tech). The tip was sequentially coated with 5-nm titanium film, 50-nm cobalt film and 5-nm gold film via electron-beam deposition. It was magnetized perpendicular to the cantilever using a permanent magnet before being mounted onto the scan head unit. The MFM images were acquired in dual-pass mode. The Gwyddion image-processing package was used to align rows. Micromagnetic simulations were carried out using the Mumax3 software package, with the material parameters set as follows: exchange constant, 1.2 × 10^−12^ J m^−1^; saturation magnetization, 2.2 × 10^5^ A m^−1^. The mesh size was 2,000 × 2,000 × 3 nm^3^.

### Ferroelastic switching by an AFM tip

Mechanical switching was performed with an AFM (Asylum MFP-3D or Park systems NX10). A diamond AFM probe with a radius of <50 nm was used. The tip was calibrated with the Sader method. The switching was performed in lithography mode, in which the tip is scanned along predefined line paths under a normal loading force and lifted while transferring among paths to avoid back-switching.

### SOT magnetization switching

Current-induced magnetization switching experiments were performed in a SrRuO_3_ (001) film of 10 nm thickness with a 4-unit-cell-thick capping layer of SrTiO_3_, patterned into a 5-μm by 5-μm Hall bar. Experiments were performed at 70 K under zero magnetic-field with a d.c. pulsed current with a duration of 100 μs applied by a Keithley 6221 current source. After each pulse, the anomalous Hall resistance (*R*_*xy*_) was measured with a 0.5-mA d.c. current. The spin current generated due to the spin Hall effect applies SOT to the magnetization, resulting in magnetization switching. This effect is described by the following equation^38^:

1 $${{\tau }}_{\mathrm{SOT}}={{\tau }}_{\mathrm{DL}}{M}\times ({M}\times {\sigma })+{{\tau }}_{\mathrm{FL}}{M}\times {\sigma }$$

where *M* is the magnetization and *σ* the spin polarization. The SOT efficiency *ξ* was calculated as 9.54 from the following formula^39,40^:

2 $$\xi =\frac{4e}{\uppi \hslash }M_{\mathrm{s}}\times t\frac{H_{\mathrm{c}}}{J_{\mathrm{SW}}}$$

where *M*_s_ is the saturated magnetization value, *t* is the thickness of the film, *H*_c_ is the coercive field in the out-of-plane direction and *J*_SW_ is the switching current density. For our calculation, we adopted the well-known magnetization value of high-quality SrRuO_3_ thin films, that is, 1.5 *μ*_B_/Ru at 10 K, along with the temperature-dependent magnetization change rate^41^.

### Numerical analysis of the contact stress

The distribution of stresses and strains induced by an AFM tip can be calculated using the Hertzian contact model and Boussinesq’s equation^42,43^. The AFM tip can be treated as a spherical indenter with a radius *R*. In cylindrical coordinates, with the centre of the contact area as the origin, the stress distribution can be determined through the superposition of Boussinesq’s solution as follows:

$$\begin{array}{rcl}\displaystyle\frac{{\sigma }_{r}(r,z)}{p}&=&\displaystyle\frac{3}{2}\left\{\displaystyle\frac{(1-2v){a}^{2}}{3{r}^{2}}\left[1-{\left(\displaystyle\frac{z}{\sqrt{u}}\right)}^{3}\right]\right.\\ && +{\left(\displaystyle\frac{z}{\sqrt{u}}\right)}^{3}\displaystyle\frac{{a}^{2}u}{{u}^{2}+{(az)}^{2}}+\displaystyle\frac{z}{\sqrt{u}}\vphantom{\frac{a}{\sqrt{u}}}\left[\displaystyle\frac{u(1-v)}{{a}^{2}+u}\right.\\ && \left.\left. +\displaystyle\frac{\left(1+v\right)\sqrt{u}}{a}{\tan }^{-1}\left(\displaystyle\frac{a}{\sqrt{u}}\right)-2\right]\right\},\end{array}$$

$$\begin{array}{l}\displaystyle\frac{{\sigma }_{\theta }(r,z)}{p}=-\displaystyle\frac{3}{2}\left\{\displaystyle\frac{(1-2v){a}^{2}}{3{r}^{2}}\left[1-{\left(\displaystyle\frac{z}{\sqrt{u}}\right)}^{3}\right]\right.\\\qquad\qquad\left.+\displaystyle\frac{z}{\sqrt{u}}\left[2v+\displaystyle\frac{u(1-v)}{{a}^{2}+u}-\displaystyle\frac{(1+v)\sqrt{u}}{a}{\tan }^{-1}\left(\displaystyle\frac{a}{\sqrt{u}}\right)\right]\right\},\end{array}$$

$$\frac{{\sigma }_{z}(r,z)}{p}=-\frac{3}{2}{\left(\frac{z}{\sqrt{u}}\right)}^{3}\frac{{a}^{2}u}{{u}^{2}+{(az)}^{2}},$$

$$\frac{{\sigma }_{rz}(r,z)}{p}=-\frac{3}{2}\left(\frac{r{z}^{2}}{{u}^{2}+{(az)}^{2}}\right)\frac{{a}^{2}\sqrt{u}}{{a}^{2}+u},$$

where *σ*_*r*_, *σ*_*θ*_ and *σ*_*z*_ denote the normal stresses, and *σ*_*rz*_ represents the shear stress. Here, *p* is the mean pressure for the contact area under the AFM tip, *v* = 0.33 and *Y* = 161 GPa are the Poisson’s ratio and Young’s modulus of the film^44,45^, *a* is the contact radius, and *u* is a quantity defined as $u=\frac{({r}^{2}+{z}^{2}-{a}^{2})+\sqrt{{({r}^{2}+{z}^{2}-{a}^{2})}^{2}+{(2az)}^{2}}}{2}$. The contact radius *a* and the mean pressure *p* are obtained as $a={(\frac{3{F}_{\mathrm{L}}R}{4{Y}^{\ast }})}^{1/3}$ and $p=\frac{{F}_{\mathrm{L}}}{\uppi {a}^{2}}$, respectively, where *F*_L_ is the loading force of the AFM tip. The effective Young’s modulus ${Y}^{\ast }={\{\frac{1-{v}^{2}}{Y}+\frac{1-{{v}_{\mathrm{tip}}}^{2}}{{Y}_{\mathrm{tip}}}\}}^{-1}$ is calculated using Poisson’s ratio and Young’s modulus for both the film and the AFM tip, with *v*_tip_ = 0.1 and *Y*_tip_ = 1,100 GPa.

Using these values and Hooke’s law, the strain components are given as:

$${\varepsilon }_{r}(r,z)=\frac{{\sigma }_{r}-\nu ({\sigma }_{\theta }+{\sigma }_{z})}{Y},\,{\varepsilon }_{z}(r,z)=\frac{{\sigma }_{z}-\nu ({\sigma }_{r}+{\sigma }_{\theta })}{Y},\,{\varepsilon }_{rz}(r,z)=\frac{{\sigma }_{rz}}{G},$$

where *G*, the shear modulus, is expressed as $G=\frac{Y}{2(1+\nu )}$. The calculated stress and strain distributions are presented in Extended Data Fig. 1, where *σ*_11_, *σ*_33_, *σ*_13_, *ε*_11_, *ε*_33_ and *ε*_13_ correspond to *σ*_*r*_, *σ*_*z*_, *σ*_*rz*_, *ε*_*r*_, *ε*_*z*_ and *ε*_*rz*_, respectively. Clearly, the tip induces large shear stress (*σ*_13_) and strain (*ε*_13_), extending spatially beyond a lateral scale of 10 nm.

When the tip scans across the surface, a tangential friction is imposed on the film surface. As a first approximation, this friction introduces an additional shear stress along the surface, proportional to the normal stress, such that *σ*_13_(**r**) = *k*_f_ *σ*_33_(**r**), where *k*_f_ denotes the friction coefficient and takes the value of 0.15 (ref. ^46^). Under these conditions, the distribution of the total shear stress and strain in the film under the sliding tip becomes distorted and asymmetric (Extended Data Fig. 2), which is consistent with previous numerical simulation results^47^. In particular, the shear stress in front of the tipʼs motion is enhanced in magnitude, while the shear stress trailing behind the tip is reduced. However, ferroelastic switching induced by the scanning tip is primarily determined by the trailing shear stress behind the tip because the region influenced by the frontal shear stress is ultimately overridden by the trailing shear stress.

## Online content

Any methods, additional references, Nature Portfolio reporting summaries, source data, extended data, supplementary information, acknowledgements, peer review information; details of author contributions and competing interests; and statements of data and code availability are available at 10.1038/s41565-025-01950-z.

## Supplementary information

Supplementary Information Supplementary Discussion, Figs. 1–13, and references 1–3.

## Source data

Source Data Figs. 4 and 5, and Extended Data Figs. 1–3, 6, 7 and 10 Statistical source data.

## Data Availability

Source data are available on figshare^48^. Additional information and data are available from the corresponding author upon reasonable request. Source data are provided with this paper.
