# Supplementary material for: Ferroelastic writing of crystal directions in oxide thin films
Source: Nat Nanotechnol. 2025 Jun 5;20(9):1199–204. doi: 10.1038/s41565-025-01950-z (PMC12443622; doi:10.1038/s41565-025-01950-z)
Supplement: Supplementary file 1 — Supplementary Discussion, Figs. 1–13, and references 1–3. [file 41565_2025_1950_MOESM1_ESM.pdf]

nature nanotechnology

Supplementary information https://doi.org/10.1038/s41565-025-01950-z

Ferroelastic writing of crystal directions in oxide thin films

In the format provided by the authors and unedited

Table of Contents

Supplementary Discussion

- Spontaneous eigenstrain states of (001) $SrRuO_3$

Supplementary Figures

- Supplementary Figure 1-13

References

- Reference 1-3

Supplementary Discussion

Spontaneous eigenstrain states of (001) $SrRuO_3$

We use the $SrRuO_3$ thin film on $SrTiO_3$ (001) as an example to show that ferroelastic domains of $SrRuO_3$ can be switched by the shear stress of an AFM tip. As shown in Supplementary Fig. 5a, the monoclinic-like distortion in $SrRuO_3$ grown on an $SrTiO_3$ (001) substrate possesses lattice shearing toward one of the in-plane (pseudo)cubic axes (i.e., x or y), resulting in a shear strain of tan0.5° ~ 0.01 (ref. 1). Besides, the lattice mismatch between $SrRuO_3$ and $SrTiO_3$ introduces epitaxial strains, with the transverse strain ε_{11} and longitudinal strain ε_{33} fixed at $\varepsilon_{11} = -0.006$ and $\varepsilon_{33} = +0.006$ for all four ferroelastic variants. Notably, these and values do not change during ferroelastic switching. Thus, we can define four degenerate spontaneous strain states: (ε_{13}, ε_{23}) = (+0.01, 0), (–0.01, 0), (0, +0.01), and (0, –0.01), corresponding to the four orientation states (Supplementary Fig. 3b). Clearly, the tip-induced shear strain is considerably larger than the spontaneous strain of ferroelastic domains in $SrRuO_3$, which suggests the potential of an AFM tip for ferroelastic switching. However, the actual value of the threshold stress for switching requires future in-depth studies.

Supplementary Figures

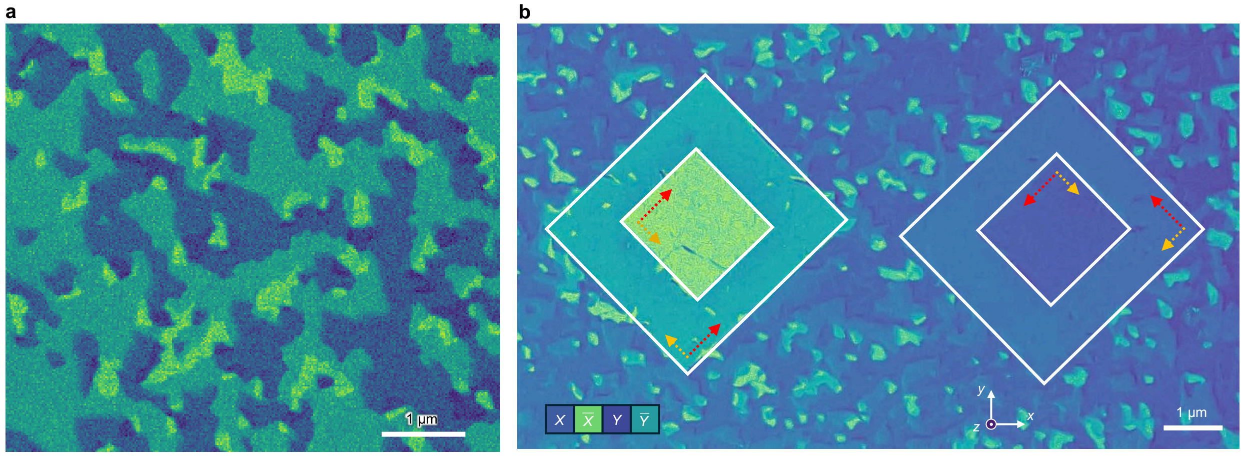

Supplementary Fig. 1. **a**, ECC image of an as-grown $(La_{0.7}Sr_{0.3})(Mn_{0.9}Ru_{0.1})O_3$ film on $(LaAlO_3)_{0.3}(Sr_2TaAlO_6)_{0.7}$ (001) substrates. The image was processed in false colors following Fig. 1b in the main text. **b**, ECC image of the $(La_{0.7}Sr_{0.3})(Mn_{0.9}Ru_{0.1})O_3$ (001) film after mechanically writing in sliding directions for four different domains. Red and yellow dashed arrows indicate fast and slow scan directions, respectively.

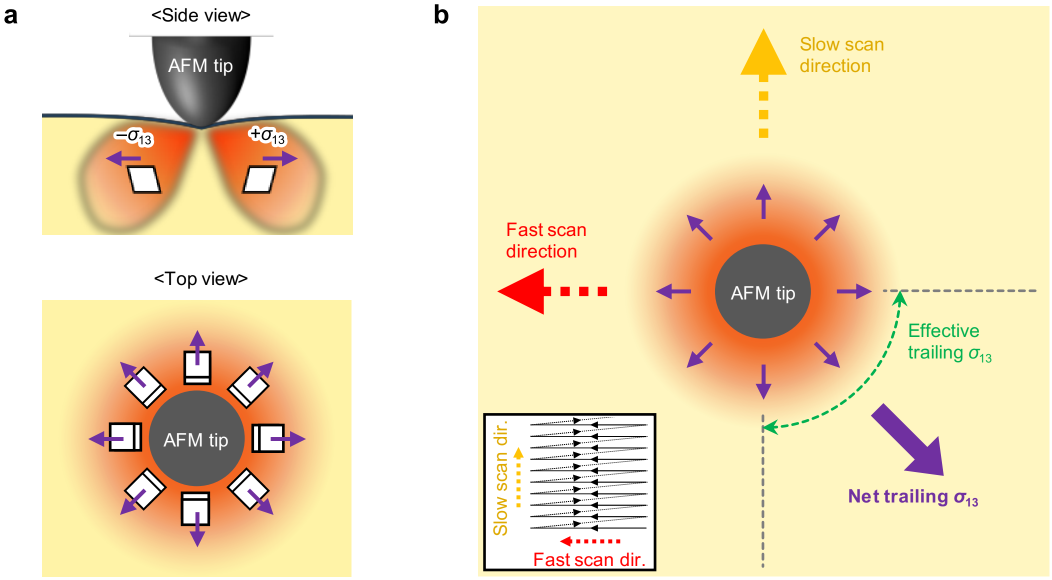

Supplementary Fig. 2. a, In the static case, an AFM tip generates shear stress σ_{13} in all directions in the vicinity of the tip contact area. **b**, In the dynamic case as the tip slides on the sample surface, effective trailing force arises according to the fast and slow scan directions, resulting in a net trailing stress field. Inset shows the trajectory (black arrows) of the AFM tip, where the fast and slow scan directions are defined. The black solid line indicates the sliding path of the tip with a normal loading force while the black dotted line indicates transfer of the tip when lifted between paths.

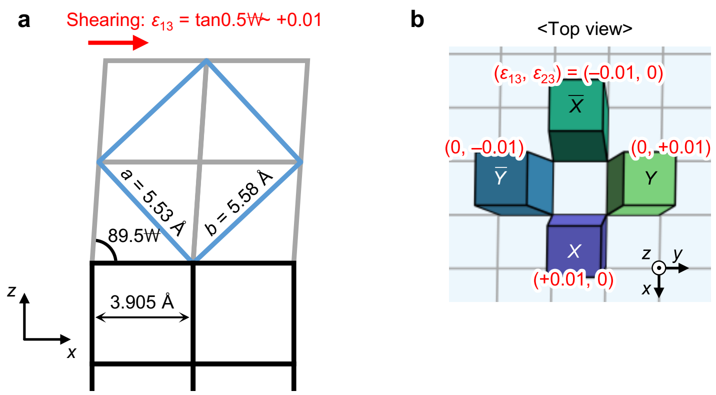

Supplementary Fig. 3. a, A schematic of $SrRuO_3$ grown on an $SrTiO_3$ (001) substrate[2]. The black, gray, and blue lines represent the cubic cell of $SrTiO_3$, and the pseudocubic and orthorhombic cells of $SrRuO_3$, respectively. The parameters *a* and *b* correspond to the short and long lattice parameters of orthorhombic $SrRuO_3$. In the pseudocubic cell, $SrRuO_3$ on $SrTiO_3$ exhibits a monoclinic-like distortion with lattice shearing of approximately 0.5°. **b**, Four avaiable ferroelastic domains in $SrRuO_3$ on $SrTiO_3$ (001), corresponding to four degenerate eigenstrain states (ε_{13}, ε_{23})

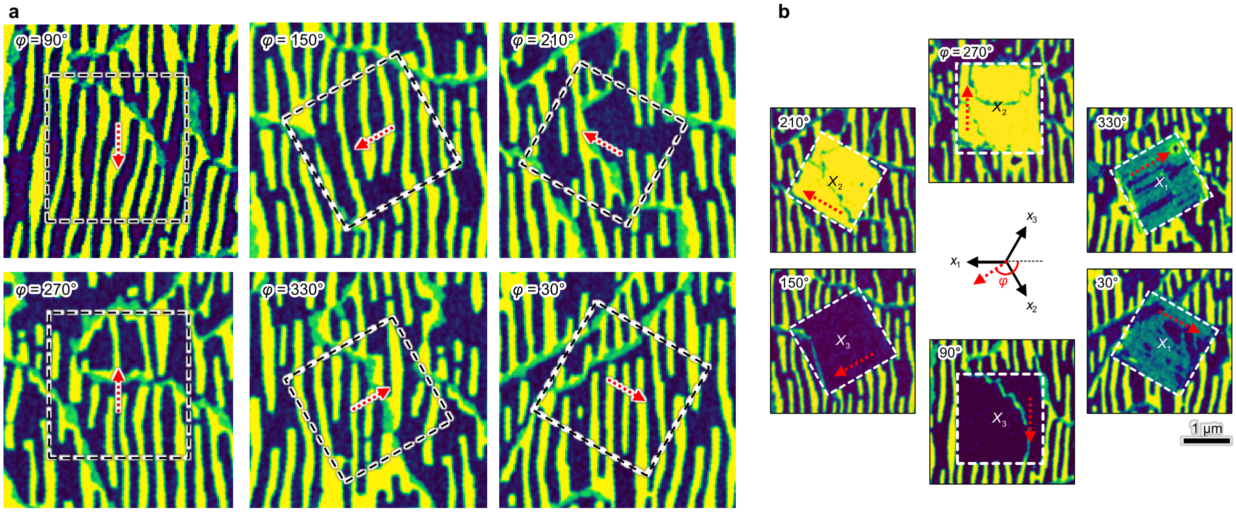

Supplementary Fig. 4. ECC images of the $SrRuO_3$ (111) film in Fig. 2 of main text before ferroelastic writing (**a**) and after writing (**b**). We note that there are contrast features in the form of curved lines throughout the images, e.g., marked by the grey arrow in (**b**), which run through the domains and refuse to be switched. We suspect that these are antiphase boundaries, which is a consequence of the lower translation symmetry of $SrRuO_3$ compared to that of the substrate.

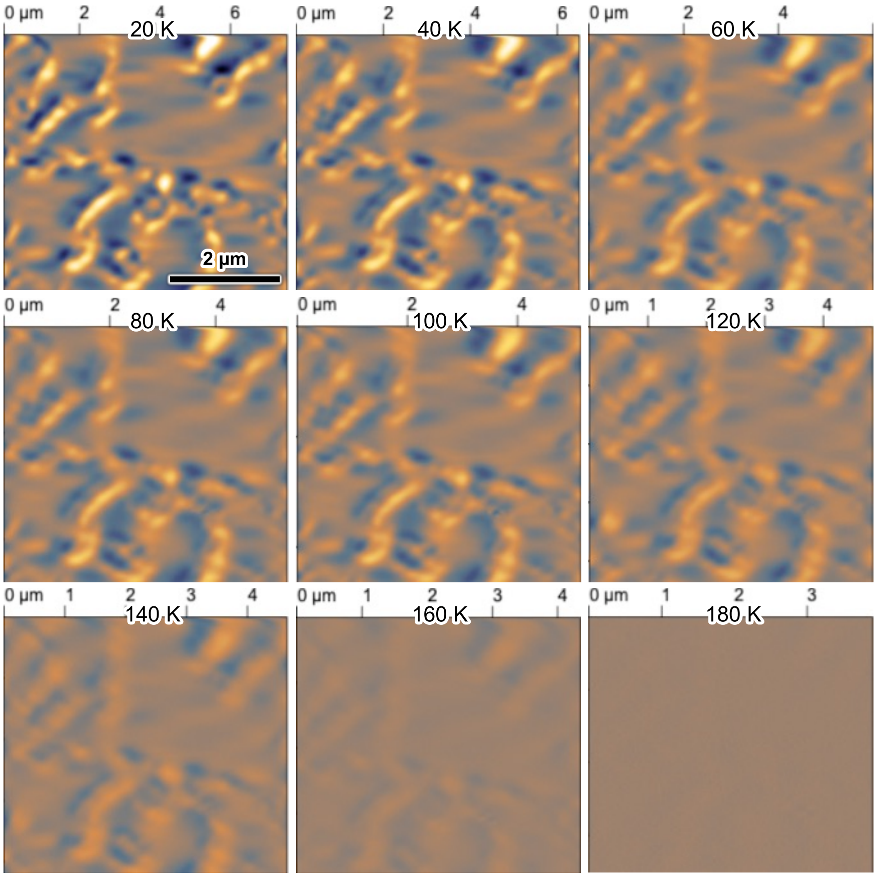

Supplementary Fig. 5. MFM images of $SrRuO_3$ (111) film acquired with a magnetic field of 1 T while heating from 20 K. The contrast vanishes near the Curie temperature (~160 K), indicating the magnetic nature of the contrast.

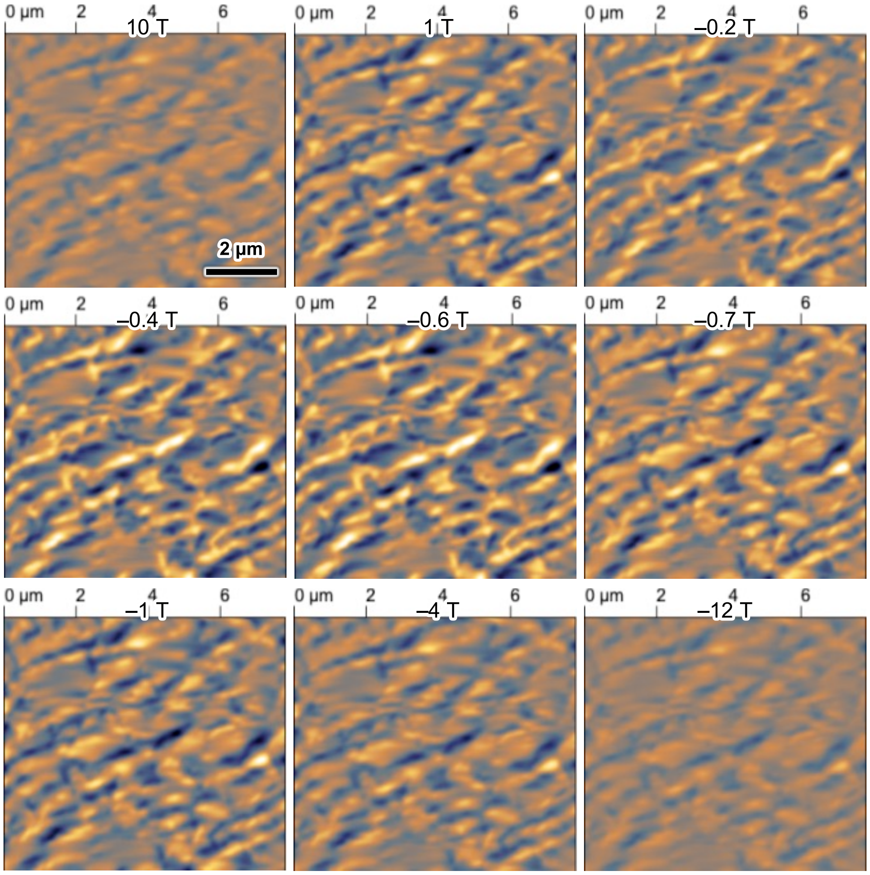

Supplementary Fig. 6. MFM images captured at 5 K with the magnetic field sweeping from 10 to –12 T, which show the robustness and spatial immobility of the magnetic contrast. Ferromagnetic switching was found to happen between –0.6 and –0.7 T.

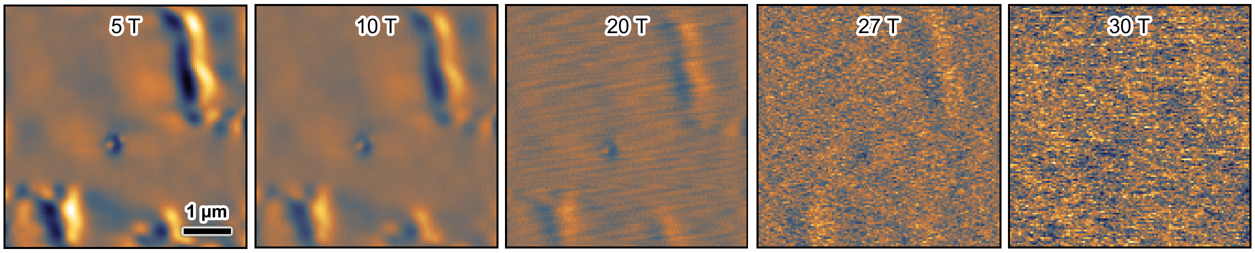

Supplementary Fig. 7. MFM images captured at 5 K with the field ramping from 5 to 30 T, which shows the vanishment of magnetic contrast above 27 T.

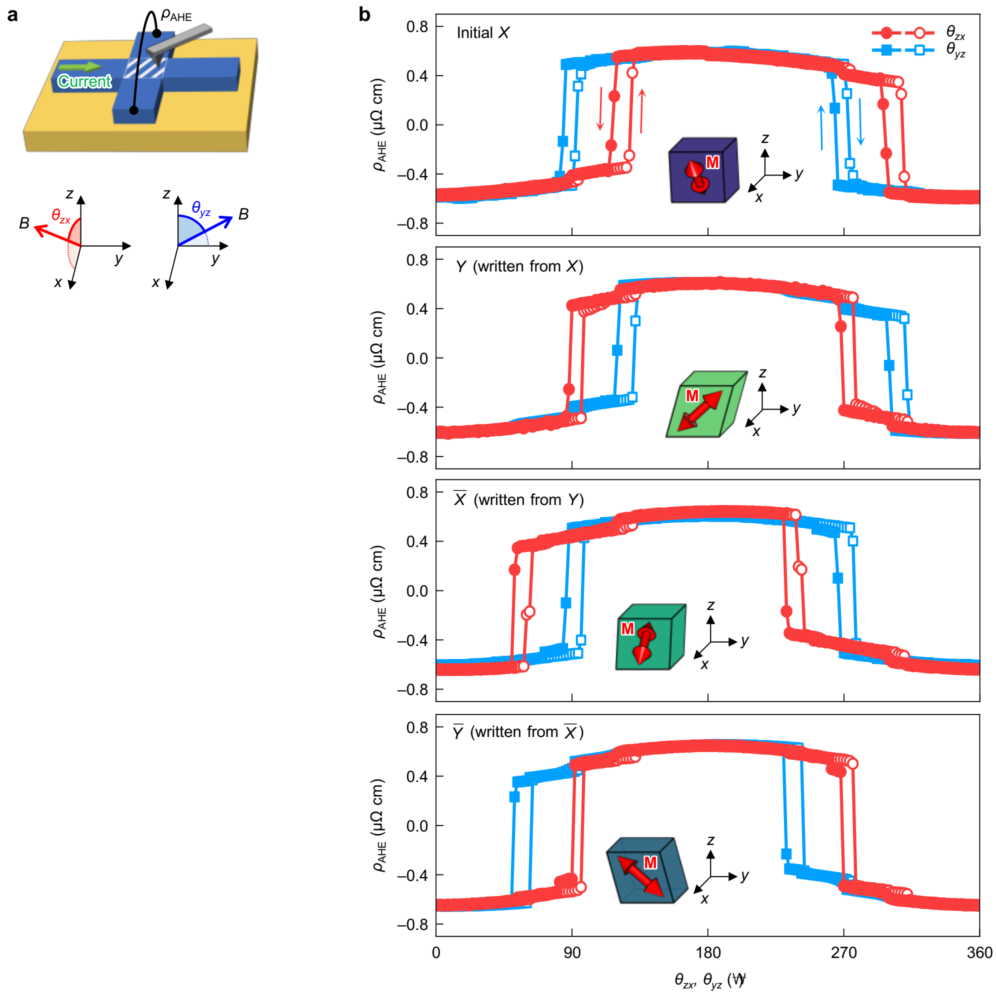

Supplementary Fig. 8. AHE results of (001) $SrRuO_3$ in Fig. 4 after consecutive ferroelastic writing of four different crystal directions.

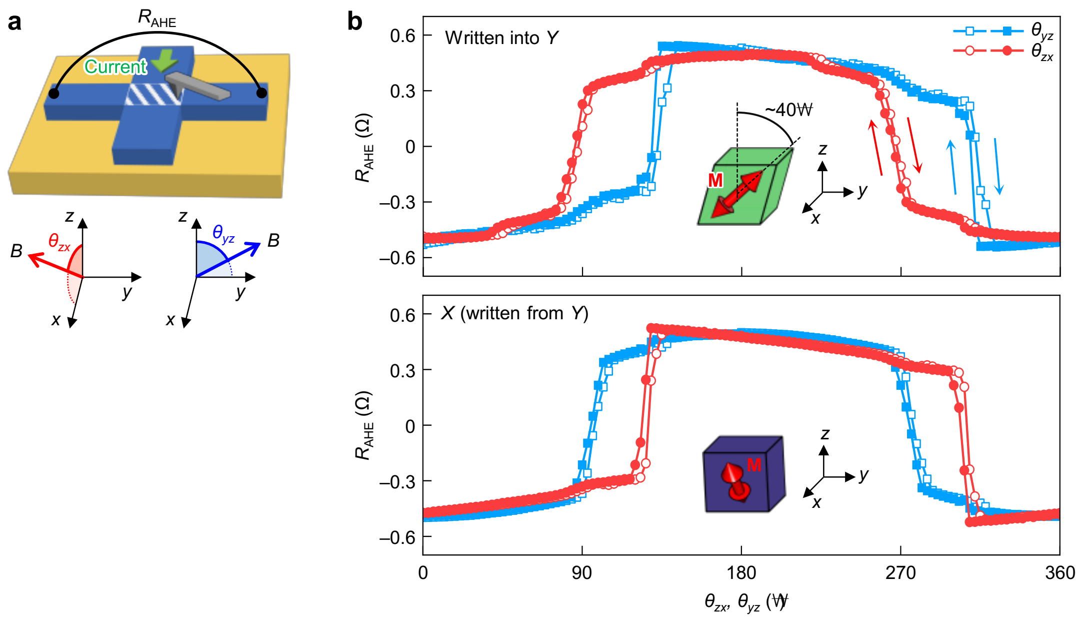

Supplementary Fig. 9. **a**, Schematic of the $(La_{0.7}Sr_{0.3})(Mn_{0.9}Ru_{0.1})O_3$ (001) Hall bar device. After ferroelastic writing with an AFM tip, anomalous Hall resistance (R_{AHE}) was measured at $T = 200$ K and $B = 0.25$ T for different field directions. θ_{zx} and θ_{yz} denote the rotation angles of the applied B field in the zx and yz planes, respectively. **b**, R_{AHE} for the mechanically written Y and X domains in a 15 nm-thick $(La_{0.7}Sr_{0.3})(Mn_{0.9}Ru_{0.1})O_3$ (001) thin film, measured as a function of θ_{zx} and θ_{yz} at $T = 200$ K and $B = 0.25$ T. The ferromagnetic coercivity of $(La_{0.7}Sr_{0.3})(Mn_{0.9}Ru_{0.1})O_3$ is relatively low, so the experiment was conducted using a small B field. Measurements were performed on the same Hall bar device after successive ferroelastic switching.

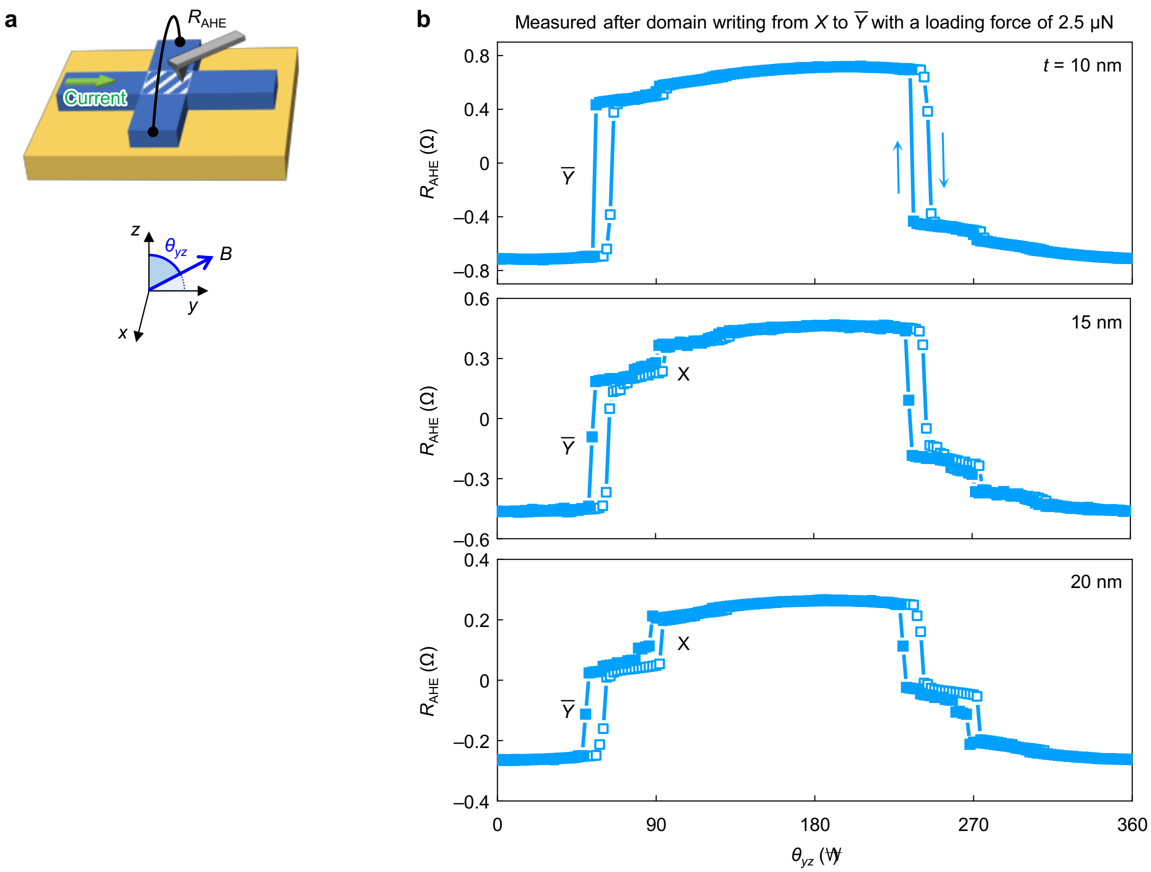

Supplementary Fig. 10. **a**, Schematic of the measurement geometry with a $SrRuO_3$ (001) Hall bar device. After ferroelastic writing, R_{AHE} was measured at $T = 70$ K and $B = 1.6$ T. θ_{yz} denote the rotation angles of the applied B field in the yz plane. **b**, Hall bar devices of $SrRuO_3$ (001) thin films with different thicknesses t (10, 15, and 20 nm) were ferroelastically written using a fixed loading force of 2.5 μN.

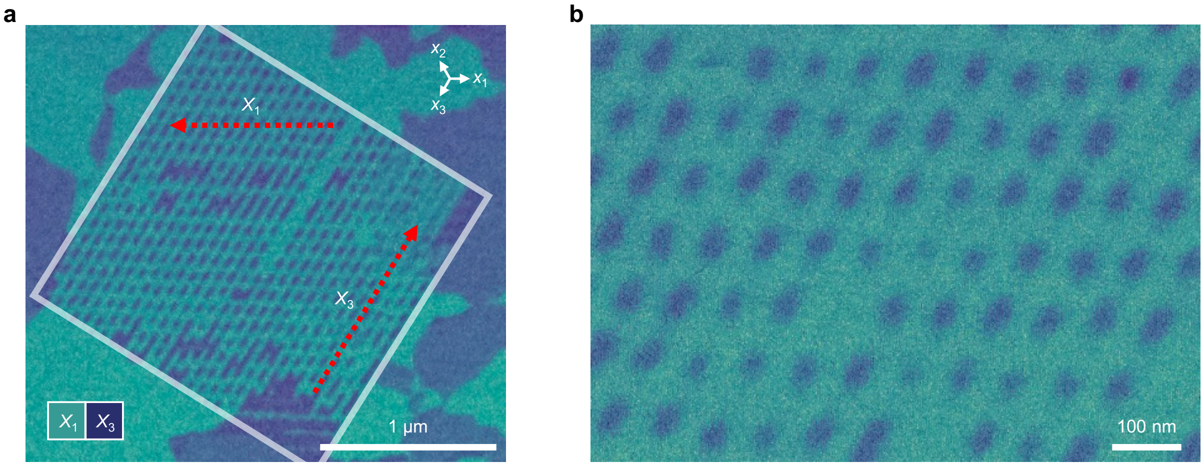

Supplementary Fig. 11. **a**, ECC image of dot domains written in a $SrRuO_3$ (111) film, which were generated by first writing a background of X_1 domain, followed by writing X_3 line domains and then X_1 line domains. This resulted in X_3 dot domains of approximately 26×26 nm^2 in size. The white box marks the region of ferroelastic writing. The red dotted arrow denotes the fast scan direction. **b**, Enlarged view of a selected region in (a), showing the dot domains.

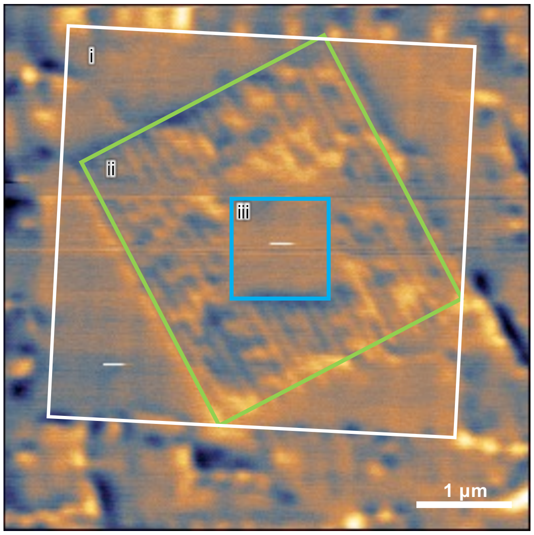

Supplementary Fig. 12. Larger-area MFM image of the artificial ferroelastic domains in Fig. 5a in the main text.

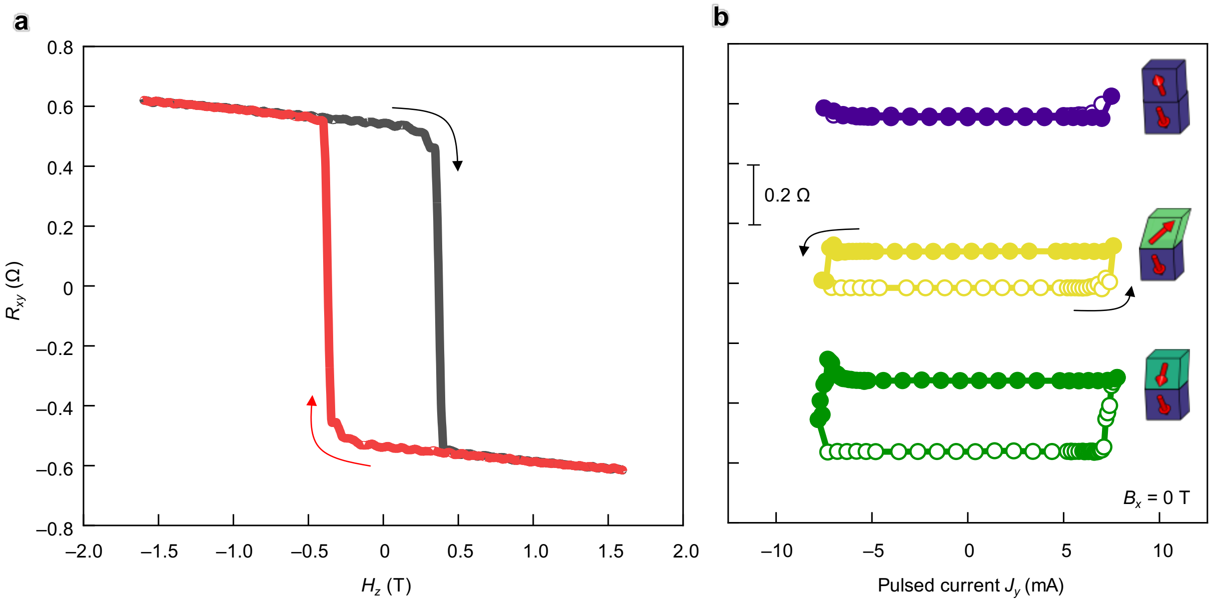

Supplementary Fig. 13. a, AHE loop measured at 70 K for 10 nm-thick $SrRuO_3$ (001) film with 4 unit cell-thick $SrTiO_3$ capping layer. The anomalous term was extracted by subtracting the linear ordinary term from ρ_{xy}. **b**, Field-free spin orbit torque (SOT) switching loops, measured at 70 K, for differently designed vertical heterogeneities of ferromagnetic anisotropy. Referring to the full AHE loop in (a), the switching ratios were found to be ~11% (yellow circles) and ~22% (green circles) for the vertical magnetic textures of $\overline{Y}/X$ and $\overline{X}/X$, respectively. For a homogeneous magnetic texture, we did not observe any SOT switching (purple circles). Considering the thickness (i.e., 10 nm) of the $SrRuO_3$ layer and its spin diffusion length (approximately 1.5 nm, ref. 3), the measured extent of partial SOT switching for each vertical magnetic texture is quantitatively reasonable.

References:

[1] R. Resta, Rev. Mod. Phys. **66**, 899 (1994).

[2] G. Koster *et al.*, Rev. Mod. Phys. **84**, 253 (2012).

[3] M. Wahler *et al.*, *Scientific Reports* **6**, 28727 (2016).
